# Comparison of Six-Month Efficacy and Safety of Rituximab in Elderly and Younger Patients With Rheumatoid Arthritis: A Prospective Real-World Study

**DOI:** 10.7759/cureus.112249

**Published:** 2026-07-08

**Authors:** Ikram El Moubarik, Imane El Binoune, Samira Rostom, Ilham Benmarzouk, Ghizlane Ghoullam, Salma Zemrani, Bouchra Amine, Zhor Zeghari, Rachid Bahiri

**Affiliations:** 1 Department of Rheumatology, El Ayachi Hospital, Centre Hospitalo-Universitaire Ibn Sina (CHUIS), Rabat, MAR; 2 Department of Public Health, Laboratory of Community Health, Preventive Medicine, and Hygiene, Faculty of Medicine and Pharmacy, Mohammed V University, Rabat, MAR; 3 Laboratory of Biostatistics, Clinical Research, and Epidemiology, Faculty of Medicine and Pharmacy, Mohammed V University, Rabat, MAR

**Keywords:** elderly patients, rheumatoid arthritis, rituximab, safety, treatment efficacy

## Abstract

Introduction

The efficacy and safety of biologic disease-modifying antirheumatic drugs, particularly tumor necrosis factor (TNF) inhibitors (anti-TNF agents), are well established in rheumatoid arthritis (RA). However, treating older patients remains challenging because of the higher burden of comorbidities, polypharmacy, and increased susceptibility to treatment-emergent adverse events (TEAEs). Rituximab (RTX), a monoclonal antibody targeting CD20-positive B cells, is widely used in our setting as a first-line biologic therapy in patients with severe disease and/or contraindications to anti-TNF agents. Nevertheless, data regarding its efficacy and safety in elderly patients remain scarce. The primary objective of this study was to compare the six-month efficacy of RTX between elderly (≥65 years) and younger (<65 years) patients with RA. Secondary objectives were to compare remission rates, low disease activity (LDA), functional outcomes, inflammatory markers, and safety between the two age groups.

Methods

This prospective real-world study included patients with RA treated with RTX, regardless of the number of previous treatment courses. The study population was stratified into an elderly group (≥65 years, Group A, n = 23) and a younger group (<65 years, Group B, n = 73). Patients underwent clinical and laboratory assessments at baseline and at four and six months following the RTX treatment cycle included in the study. Therapeutic response was determined using changes in the Disease Activity Score in 28 joints calculated with CRP (DAS28-CRP), as well as remission and LDA rates. The primary outcome was the change in DAS28-CRP from baseline to month 6. Safety was assessed by recording TEAEs, including infections, serious TEAEs, and treatment discontinuation.

Results

A total of 96 patients were included: 23 elderly patients (mean age 69.4 ± 2.8 years; 78.3% female) and 73 younger patients (mean age 53.2 ± 7.1 years; 89.0% female). Baseline demographic and disease characteristics were generally comparable between groups. Significant improvement in disease activity was observed in both groups at four and six months. At six months, the mean improvement in DAS28-CRP was -2.17 (95% CI: -2.82 to -1.41) in elderly patients and -2.27 (95% CI: -2.59 to -1.75) in younger patients (p = 0.90). Remission rates were similar between elderly and younger patients (11/23 (47.8%) vs. 31/73 (42.5%), p = 0.81). TEAEs were uncommon in both groups and consisted mainly of mild infections, with no treatment-related deaths reported.

Conclusions

This prospective real-world study suggests that RTX was associated with similar short-term efficacy outcomes and a low incidence of TEAEs in elderly and younger patients with RA. In this study, advanced age was not associated with a poorer therapeutic response to RTX. These findings provide reassuring short-term real-world evidence supporting the use of RTX in older adults with RA within an individualized treatment strategy. Larger multicenter studies are warranted to confirm these findings.

## Introduction

Rheumatoid arthritis (RA) is a chronic inflammatory rheumatic disease that affects adults and may result in progressive joint damage, physical dysfunction, and considerable long-term disability if inadequately controlled. Nearly 30% of patients with RA are aged 65 years or older, and this proportion is expected to increase with population aging and improved life expectancy [1]. The prevalence of elderly-onset RA (EORA) is expected to rise, posing a growing clinical and therapeutic challenge.

EORA is typically characterized by a relatively abrupt clinical presentation and may be associated with constitutional symptoms such as fatigue, fever, and unintentional weight loss [1-3]. Approximately 70% of patients present with a clinical pattern similar to that of younger-onset RA, while around 25% show predominant involvement of the shoulder and pelvic girdles, and a smaller proportion (about 5%) present with symmetric, seronegative, relapsing synovitis with pitting edema [4].

The disease course in elderly patients appears to be heterogeneous. Some studies have reported a milder prognosis, whereas others have shown similar or even more aggressive progression compared with that in younger adults [2]. Management of RA in older adults is further complicated by age-related comorbidities and an increased risk of drug-related adverse events. Medications such as corticosteroids, methotrexate, and biologics often demonstrate reduced tolerability in this population [5].

Despite these challenges, the therapeutic goals remain consistent across age groups: controlling clinical symptoms, preventing radiographic progression, maintaining physical function, and reducing mortality. Available evidence indicates that conventional antirheumatic therapies and biologic agents targeting tumor necrosis factor (TNF) have improved disease outcomes in patients with RA and are generally effective and well tolerated in older patients [6-8].

Rituximab (RTX), a chimeric monoclonal antibody targeting CD20-positive B cells, was initially developed for the treatment of non-Hodgkin lymphoma and is now widely used in RA. Although generally considered relatively safe, RTX can cause immunosuppression through B-cell depletion and hypogammaglobulinemia, thereby increasing the risk of infections. For this reason, regular monitoring of lymphocyte counts and serum IgG levels is recommended [9,10].

Significant progress in the management of RA has been achieved through the use of conventional therapeutic agents and biologic drugs that interfere with TNF-mediated inflammatory pathways. In routine clinical practice, off-label use of RTX as a first-line biologic therapy may occasionally be considered according to individual patient characteristics and contraindications [11].

Despite its increasing use in routine clinical practice, data regarding the efficacy and safety of RTX specifically in elderly patients with RA remain limited. To our knowledge, only three studies have specifically investigated this population [4,12,13]. Given their potentially increased susceptibility to infectious complications, elderly patients require careful evaluation and monitoring. This issue is particularly relevant in Morocco, where RTX has become one of the most frequently prescribed biologic therapies for RA.

The primary objective of this study was to compare the six-month real-world effectiveness of RTX between elderly (≥65 years) and younger (<65 years) patients with RA. Secondary objectives were to compare remission rates, low disease activity (LDA), functional outcomes, inflammatory markers, and treatment-emergent adverse events (TEAEs) between the two age groups.

## Materials and methods

Study design

We conducted a single-center prospective observational study involving patients with RA followed in the Rheumatology Department of Ibn Sina University Hospital, Rabat, Morocco, between August 2023 and March 2025. Ethical approval was obtained from the local ethics committee (CERB 144-25), and the study was conducted in accordance with the principles of the Declaration of Helsinki. Written informed consent was obtained from all participants before the collection and analysis of study data.

Clinical and laboratory data were prospectively collected during standardized follow-up visits using predefined case report forms and were subsequently verified through review of the electronic medical records before statistical analysis. As this was a prospective observational study, all consecutive patients who met the eligibility criteria and received RTX during the study period were included. Owing to the exploratory nature of the study, no formal sample size calculation was performed. The sample size was therefore determined by the number of eligible patients available during the study period and was intended to provide real-world data on the efficacy and safety of RTX in elderly patients with RA.

Inclusion and exclusion criteria

Patients aged 18 years or older with RA fulfilling the 2010 American College of Rheumatology/European Alliance of Associations for Rheumatology (EULAR) classification criteria were eligible for inclusion [14]. Participants were either receiving RTX or considered eligible for RTX therapy, regardless of previous exposure to conventional synthetic disease-modifying antirheumatic drugs (csDMARDs) or biologic disease-modifying antirheumatic drugs (bDMARDs). For patients who had previously received RTX, baseline (M0) was defined as the clinical and laboratory assessment performed immediately before the RTX infusion cycle included in the present study, regardless of the number of previous RTX treatment courses.

For comparative analyses, patients were classified as either younger (<65 years) or elderly (≥65 years). All participants were followed for six months after the RTX treatment cycle included in the study. Patients were excluded if they were younger than 18 years of age, had severe cognitive impairment, refused participation, or had incomplete follow-up (<6 months). No imputation of missing data was performed because patients with incomplete follow-up were excluded from the final analysis. No major protocol deviations occurred during the six-month follow-up.

Before RTX initiation, all patients underwent routine screening for hepatitis B virus, hepatitis C virus, HIV infection, and active infection, including chest radiography, according to institutional practice. RTX was administered according to routine clinical practice in our department. The initial treatment course consisted of two intravenous infusions of 1,000 mg administered two weeks apart (days 1 and 15). Retreatment courses consisted of two 500-mg infusions administered two weeks apart and were prescribed at the discretion of the treating rheumatologist based on disease activity, clinical relapse, and previous treatment response. Before each infusion, patients received standard premedication consisting of intravenous methylprednisolone (120 mg), oral paracetamol (1 g), and an antihistamine to reduce the risk of infusion-related reactions.

Outcome measures

The primary outcome was the change in the Disease Activity Score in 28 joints calculated with CRP (DAS28-CRP) between baseline (M0) and month 6 (M6) [15]. The primary endpoint was assessed using the validated four-variable DAS28-CRP formula incorporating the tender joint count (TJC; 28 joints), swollen joint count (SJC; 28 joints), patient global assessment (100-mm Visual Analogue Scale, VAS), and serum CRP concentration. Remission and LDA were defined as DAS28-CRP <2.6 and ≤3.2, respectively.

Secondary outcomes included disease activity at month 6 assessed using the Disease Activity Score in 28 joints calculated with erythrocyte sedimentation rate (DAS28-ESR) [15] and the Simplified Disease Activity Index (SDAI) [16], remission rates, LDA, functional outcomes (Health Assessment Questionnaire, HAQ [17]), inflammatory markers (CRP and erythrocyte sedimentation rate, ESR), and TEAEs.

Clinical response was assessed at month 4 (M4) and month 6 (M6) using DAS28-CRP, DAS28-ESR, SDAI, and the HAQ. Inflammatory markers, including ESR and CRP, were also assessed. TEAEs were defined as any untoward medical event occurring after RTX administration, regardless of whether it was considered causally related to treatment. Serious TEAEs were defined as events resulting in hospitalization, intensive care unit admission, or death. Safety outcomes included the occurrence of TEAEs, serious TEAEs, and treatment discontinuation due to toxicity.

Data collection

Data were collected from electronic medical records and standardized clinical assessments performed during follow-up visits. Collected variables included age, sex, and other relevant demographic characteristics; disease-related characteristics (symptom onset, date of diagnosis, anti-citrullinated protein antibody status, rheumatoid factor (RF) positivity, erosive disease, and disease severity); comorbidities (diabetes mellitus, hypertension, dyslipidemia, cardiovascular disease, osteoporosis, stroke, smoking status, menopause, depression, and fibromyalgia); and treatment-related characteristics (number of RTX cycles, dosage regimen, end-of-dose effect, concomitant corticosteroid therapy, and previous or current DMARD exposure).

Patients were evaluated at baseline (M0), month 4 (M4), and month 6 (M6). During each visit, TJC, SJC, pain VAS, patient global assessment VAS, DAS28-CRP, DAS28-ESR, SDAI, HAQ, ESR, and CRP were recorded. Baseline serum Ig levels (IgG, IgA, and IgM) were routinely measured before RTX initiation according to local clinical practice. However, systematic longitudinal Ig monitoring during follow-up was not available for all patients. Serum CRP concentrations were measured in the hospital biochemistry laboratory using a standard immunoturbidimetric assay. At each follow-up visit, patients were systematically screened for treatment-emergent infections through clinical examination, review of medical records, hospitalization records, and laboratory or microbiological investigations when clinically indicated.

Statistical analysis

Statistical analyses were performed using jamovi software, version 2.3.28 (The jamovi Project, Sydney, Australia). Continuous variables were expressed as mean ± SD or median with IQR, according to data distribution. Categorical variables were reported as numbers and percentages (n (%)). Comparisons between age groups were performed using Student’s t-test or the Mann-Whitney U test for continuous variables and the chi-square test or Fisher's exact test for categorical variables, as appropriate.

Treatment efficacy was assessed using the change in DAS28-CRP from baseline to month 6 (ΔDAS28-CRP) and the proportions of patients achieving remission or LDA at month 6. Treatment safety was evaluated by analyzing the frequency and nature of TEAEs, including serious TEAEs and events leading to treatment discontinuation. All statistical tests were two-sided, and a p-value <0.05 was considered statistically significant. Because this was an exploratory observational study with a limited number of predefined analyses, no formal adjustment for multiple comparisons was performed. Therefore, the results should be interpreted with appropriate caution.

## Results

Study population characteristics

The study population comprised 96 patients with RA, with a mean age of 57.1 ± 9.4 years. Women represented the majority of participants (83/96, 86.5%). Patients were stratified into a younger cohort (<65 years, n = 73; mean age, 53.2 ± 7.1 years; 65/73 (89.0%) female) and an elderly cohort (≥65 years, n = 23; mean age, 69.4 ± 2.8 years; 18/23 (78.3%) female). Overall, 78 patients (81.3%) had at least one comorbidity, the most common being osteoporosis (68.8%), hypertension (31.3%), and diabetes mellitus (24.0%).

The median disease duration was 16 years (IQR, 8.5-24), and 92 (95.8%) patients were positive for RF and/or anti-citrullinated protein antibodies. Erosive disease was observed in 71 (74.0%) patients. At study inclusion, 37 (38.5%) patients were not receiving csDMARDs. Among treated patients, methotrexate was prescribed in 25 (26.0%), sulfasalazine in 26 (27.1%), and leflunomide in 8 (8.3%). Corticosteroids were used by 73 (76.0%) patients, with a median dose of 5 mg/day (IQR, 2.25-6.25). Previous biologic therapy had been used in 8/96 (8.3%) patients.

Apart from the predefined difference in age resulting from the study design, baseline demographic, clinical, and treatment characteristics were generally comparable between the two groups. Hypertension was significantly more frequent among elderly patients (12/23 (52.2%) vs. 18/73 (24.7%), p = 0.013), whereas no significant differences were observed for disease duration, seropositivity, erosive disease, concomitant csDMARD use, or corticosteroid exposure (Table 1).

**Table 1:** Baseline demographic, clinical, and treatment characteristics of patients with RA stratified by age (<65 years and ≥65 years) Continuous variables are presented as mean ± SD or median (IQR), as appropriate, and categorical variables as n (%). Comparisons between groups were performed using Student’s t-test or the Mann-Whitney U test for continuous variables and the chi-square test or Fisher’s exact test for categorical variables, as appropriate. A p-value <0.05 was considered statistically significant. ^*^ Presence of at least one comorbidity csDMARDs: conventional synthetic disease-modifying antirheumatic drugs; RA: rheumatoid arthritis

Variable	Total (n = 96)	<65 years (n = 73)	≥65 years (n = 23)	p-value
Age (years)	57.1 ± 9.4	53.2 ± 7.07	69.4 ± 2.81	<0.001
BMI (kg/m²)	26 (22.9-29.9)	26 (23-30.8)	24.8 (21.8-29)	0.412
Female sex, n (%)	83 (86.5)	65 (89.0)	18 (78.3)	0.291
Comorbidities, n (%)^*^	78 (81.3)	57 (78.1)	21 (91.3)	0.224
Hypertension	30 (31.3)	18 (24.7)	12 (52.2)	0.013
Diabetes	23 (24.0)	17 (23.3)	6 (26.1)	0.784
Cardiopathy	3 (3.1)	1 (1.4)	2 (8.7)	0.142
Osteoporosis	66 (68.8)	50 (68.5)	16 (69.6)	0.544
Coxitis, n (%)	3 (3.1)	2 (2.7)	1 (4.3)	0.565
Atlanto-axial involvement, n (%)	7 (7.3)	6 (8.2)	1 (4.3)	1.000
RA duration (years)	16 (8.5-24)	16 (7-24)	17.5 (12.3-19.8)	0.494
Seropositivity, n (%)	92 (95.8)	70 (95.9)	22 (95.7)	1.000
Erosive disease, n (%)	71 (74.0)	52 (71.2)	19 (82.6)	1.000
Current csDMARDs, n (%)				0.955
No csDMARDs	37 (38.5)	29 (39.7)	8 (34.8)	
Methotrexate	25 (26.0)	18 (24.7)	7 (30.4)	
Leflunomide	8 (8.3)	6 (8.2)	2 (8.7)	
Sulfasalazine	26 (27.1)	20 (27.4)	6 (26.1)	
Corticosteroids, n (%)	73 (76.0)	58 (79.5)	15 (65.2)	0.163
Corticosteroid dose (mg/day)	5 (2.25-6.25)	5 (2.5-7.5)	5 (0-5)	0.286

RTX treatment patterns

Patients had received different numbers of RTX treatment cycles at study inclusion. Overall, 11 (11.5%) patients had received one cycle, 26 (27.1%) had received two cycles, 19 (19.8%) had received three cycles, and 40 (41.7%) had received four or more cycles. The most frequently prescribed regimen was 500 mg × 2, administered to 56 (58.3%) patients, followed by 1 g × 2 in 35 (36.5%) patients and 500 mg × 1 in five (5.2%) patients. No significant differences in dosing regimen or number of treatment cycles were observed between the two age groups (Table 2).

**Table 2:** RTX treatment patterns according to age group (<65 years vs. ≥65 years) Data are presented as n (%). Comparisons between groups were performed using the chi-square test or Fisher’s exact test, as appropriate. A p-value <0.05 was considered statistically significant. RTX: rituximab

Number of RTX cycles	Total (%), n = 96	<65 years (%), n = 73	≥65 years (%), n = 23	p-value
1 cycle	11 (11.5)	8 (11.0)	3 (13.0)	0.491
2 cycles	26 (27.1)	18 (24.7)	8 (34.8)	
3 cycles	19 (19.8)	18 (24.7)	1 (4.3)	
4 cycles	20 (20.8)	14 (19.2)	6 (26.1)	
≥ cycles	20 (20.8)	15 (20.5)	5 (21.7)	
RTX dosage				0.272
500 mg ×1	5 (5.2)	5 (6.8)	0 (0)	
500 mg ×2	56 (58.3)	44 (60.3)	12 (52.2)	
1 g ×2	35 (36.5)	24 (32.9)	11 (47.8)	

Efficacy of RTX

**Figure 1:**
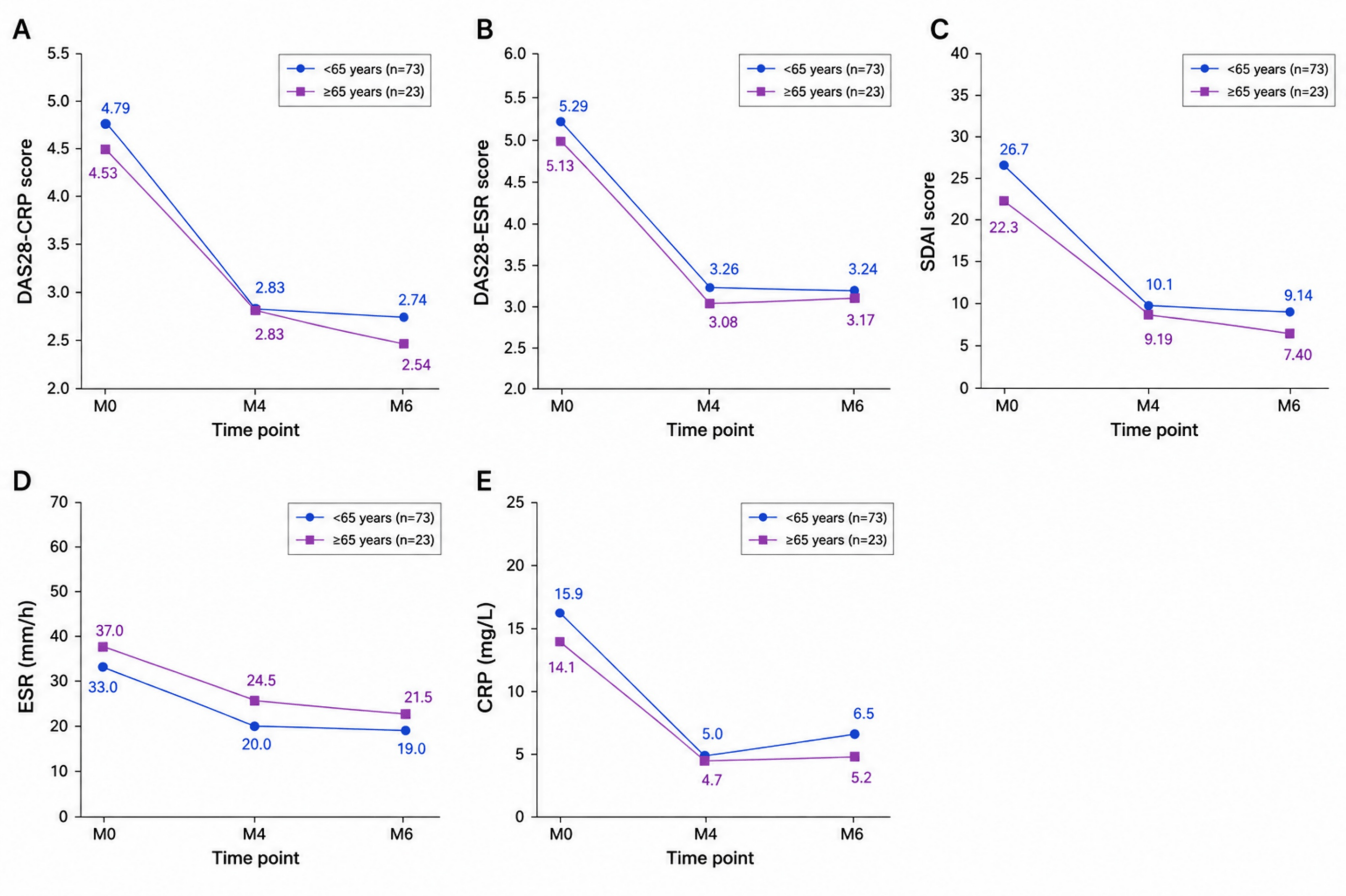
Evolution of disease activity scores and inflammatory markers under RTX therapy according to age group (A) DAS28-CRP, primary outcome measure, at baseline (M0), month 4 (M4), and month 6 (M6). (B) DAS28-ESR at M0, M4, and M6. (C) SDAI at M0, M4, and M6. (D) ESR (mm/h) at M0, M4, and M6. (E) CRP (mg/L) at M0, M4, and M6. DAS28-CRP: Disease Activity Score in 28 joints calculated with CRP; DAS28-ESR: Disease Activity Score in 28 joints calculated with erythrocyte sedimentation rate; ESR: erythrocyte sedimentation rate; RA: rheumatoid arthritis; RTX: rituximab; SDAI: Simplified Disease Activity Index

Both age groups demonstrated substantial clinical improvement throughout follow-up, and treatment responses remained similar between groups over time (Figure 1).

Baseline Evaluation (M0)

At baseline, disease activity and functional status were comparable between groups. Median DAS28-CRP was 4.79 (4.04-5.44) in younger patients and 4.53 (3.81-5.52) in elderly patients (p = 0.818). SDAI scores were 26.7 (19.5-34.0) and 22.3 (16.3-37.3), respectively (p = 0.624), while median HAQ scores were 1.0 (0.8-1.40) and 1.0 (0.8-1.65), respectively (p = 0.643). ESR and CRP levels were also comparable between groups.

Follow-Up at Month 4 (M4)

At month 4, both groups showed marked clinical and biological improvement. Median DAS28-CRP decreased to 2.83 (2.26-3.43) in younger patients and 2.83 (2.36-4.02) in elderly patients (p = 0.534). Similar improvements were observed for SDAI, HAQ, ESR, and CRP values, with no significant between-group differences.

Follow-Up at Month 6 (M6)

Continued improvement was observed at month 6. Median DAS28-CRP was 2.74 (2.04-3.68) in younger patients and 2.54 (1.94-3.30) in elderly patients (p = 0.446). DAS28-ESR, SDAI, HAQ, ESR, and CRP values remained comparable between groups.

The mean change in DAS28-CRP from baseline was -2.17 (95% CI: -2.82 to -1.41) in elderly patients and -2.27 (95% CI: -2.59 to -1.75) in younger patients (p = 0.90), suggesting similar short-term treatment responses across age groups.

**Figure 2:**
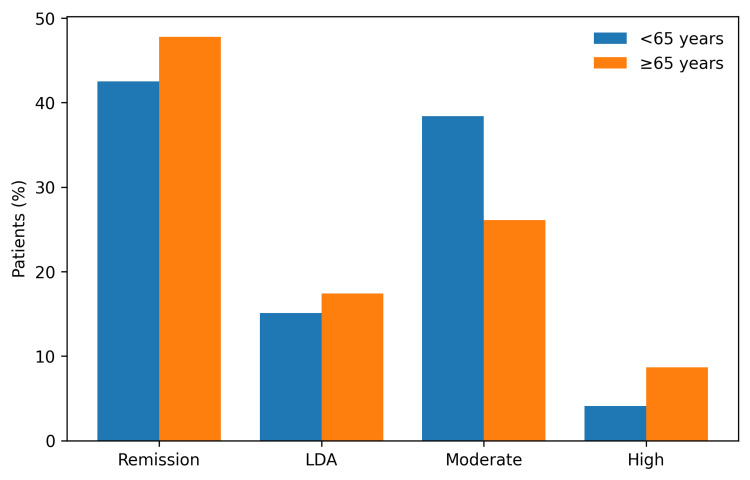
Distribution of disease activity status (DAS28-CRP) at six months according to age group DAS28-CRP: Disease Activity Score in 28 joints calculated with CRP; LDA: low disease activity

At month 6, remission (DAS28-CRP <2.6) was achieved by 31/73 (42.5%) younger patients and 11/23 (47.8%) elderly patients (p = 0.81). LDA was achieved by 11/73 (15.1%) and 4/23 (17.4%) patients, respectively (p = 0.75). Moderate disease activity persisted in 28/73 (38.4%) younger patients and 6/23 (26.1%) elderly patients. High disease activity remained in 3/73 (4.1%) younger patients and 2/23 (8.7%) elderly patients (Figure 2).

Safety and tolerability

TEAEs are summarized in Table 3. All reported adverse events were classified as TEAEs because they occurred after RTX administration. However, owing to the observational design of the study, a definite causal relationship with RTX could not be established. TEAEs were recorded in both age groups throughout the six-month follow-up period. No statistically significant difference was observed in the overall infection rate between younger and elderly patients (11/73 (15.1%) vs. 4/23 (17.4%), p = 0.751).

**Table 3:** TEAEs according to age group (<65 years vs. ≥65 years) ^*^ Three patients required hospitalization because of serious bacterial infections (two patients aged <65 years and one patient aged ≥65 years). ^†^ Bacterial infections included septic monoarthritis, tuberculous spondylodiscitis, pneumonia, UTIs, and gastroenteritis. RTX: rituximab; TEAE: treatment-emergent adverse event

Adverse event	<65 years (n = 73)	≥65 years (n = 23)	Predominant severity	Hospitalizations, n	p-value
Any infection	11 (15.1%)	4 (17.4%)	Mostly mild	3 patients^*^	0.751
Viral infections (influenza or COVID-19)	6 (8.2%)	3 (13.0%)	Mild	No	-
Bacterial infections^†^	5 (6.8%)	1 (4.3%)	Mild-severe	Yes (3 cases)^*^	-
Serious adverse events (hospitalization)	2 (2.7%)	1 (4.3%)	Severe	Yes	0.565
Treatment discontinuation due to toxicity	0	0	-	-	-
Death	0	0	-	-	-

Most infections were mild and managed on an outpatient basis. Viral infections, including influenza and COVID-19, occurred in 6/73 (8.2%) younger patients and 3/23 (13.0%) elderly patients. Bacterial infections were reported in 5/73 (6.8%) younger patients and 1/23 (4.3%) elderly patients. These included septic monoarthritis, tuberculous spondylodiscitis, pneumonia, UTIs, and gastroenteritis.

Three serious adverse events requiring hospitalization occurred during follow-up, including two among younger patients (2/73, 2.7%) and one among elderly patients (1/23, 4.3%) (p = 0.565). No deaths occurred during the study period, and no patient permanently discontinued RTX because of treatment-related toxicity.

Overall, no statistically significant differences were observed between age groups in the frequency of TEAEs. However, given the limited number of elderly patients and the low number of serious adverse events, these findings should not be interpreted as evidence of equivalent safety.

## Discussion

In elderly patients, the management of immune-mediated diseases must be optimized with the same rigor as in younger individuals. However, age-specific challenges such as frailty, polypharmacy, and comorbid conditions require particular attention. Notably, there is a tendency to under-prescribe bDMARDs in older adults because of concerns regarding safety and tolerability [18]. To ensure appropriate use, each bDMARD should be preceded by a molecule-specific pretreatment workup, with thorough screening for comorbidities that are more commonly encountered in this population.

The growing availability of bDMARDs offers new therapeutic possibilities but also presents potential risks, particularly in elderly patients. These treatments can result in serious adverse events, warranting comprehensive pretreatment evaluation and adherence to established contraindications and precautions. For clinical decision-making, existing recommendations, such as those from the VIDAL drug compendium [19] and the Club Rhumatismes et Inflammations (CRI), provide valuable guidance, especially regarding the most commonly used biologics [20].

Among bDMARDs, TNF-α inhibitors remain the most extensively studied in older populations. For agents including infliximab, adalimumab, certolizumab, golimumab, and etanercept, pharmacokinetic studies have not demonstrated significant differences in serum concentrations or drug clearance in individuals aged over 65 years, indicating no need for age-related dose adjustment [19]. While some studies suggest comparable efficacy between age groups, others report a diminished response in older adults [21,22]. Tolerability appears to be either equivalent or somewhat reduced in older patients, largely due to age-associated factors rather than the biologic agents themselves [23].

Efficacy data from registries and clinical studies

RTX is a biologic therapy that selectively targets CD20-expressing B cells and has demonstrated efficacy in both autoimmune diseases and hematological malignancies. In RA, RTX is an established therapeutic option; however, data regarding its use in elderly patients remain limited because this population has historically been underrepresented in clinical trials [4,12,13].

Published data on RTX efficacy in elderly patients with RA have yielded inconsistent findings. In the French AIR registry, Payet et al. reported a trend toward lower EULAR response rates and significantly higher DAS28-ESR scores after one year of treatment in patients older than 75 years [4]. Conversely, the German GERINIS registry did not identify significant differences in treatment response between older and younger patients [13]. Similarly, Mielnik et al. found no association between age and treatment effectiveness or persistence, although pneumonia occurred more frequently among elderly individuals [12]. These heterogeneous findings highlight the complexity of assessing treatment response in older patients and emphasize the need for additional real-world studies.

Infectious risks and tolerability: predictive factors and biological rationale

The risk of infection remains a major concern during RTX therapy, particularly in elderly patients. Through B-cell depletion, RTX may induce hypogammaglobulinemia and impair humoral immunity, thereby increasing susceptibility to infections [24].

In the AIR registry, Gottenberg et al. identified advanced age, extra-articular disease manifestations, and low baseline IgG levels as independent predictors of serious infections in patients receiving RTX [24]. Additional risk factors include chronic lung disease, heart failure, RTX-induced neutropenia, and exposure to high-dose corticosteroids [25].

Although advanced age has been associated with adverse outcomes in several studies, current recommendations from the French CRI do not advocate RTX dose adjustment solely on the basis of age [20]. Supporting this position, Boleto et al. found no significant association between age ≥65 years and the development of hypogammaglobulinemia during RTX treatment [26].

Data from the DANBIO registry further suggest that, although infection rates may be higher during the first year of RTX treatment compared with abatacept or tocilizumab, the absolute risk of infection remains relatively stable across different inflammatory rheumatic diseases [27]. In addition, a meta-analysis by Castagné et al. found no increased risk of cardiovascular events with RTX compared with tocilizumab, further supporting the overall safety profile of RTX in older patients [28].

Our study findings in context

This prospective real-world study adds to the limited evidence by comparing the short-term efficacy and safety of RTX between elderly and younger patients with RA.

Both groups showed significant clinical and functional improvement after six months of treatment, as reflected by parallel reductions in DAS28-CRP, DAS28-ESR, SDAI scores, inflammatory markers, and HAQ scores. The magnitude of DAS28-CRP reduction was similar between age groups, suggesting that elderly patients experienced short-term clinical improvement comparable to that observed in younger patients.

Similarly, remission rates were comparable between elderly patients (11/23, 47.8%) and younger patients (31/73, 42.5%). These results are consistent with findings from the GERINIS registry, which also did not observe a significant impact of age on long-term treatment efficacy [13]. In contrast, the AIR registry reported lower remission rates among older patients, suggesting that age-related factors such as immunosenescence, longer disease duration, or differing therapeutic goals may influence outcomes in some cohorts [4].

Adverse events were observed in both age groups, with no statistically significant differences in the overall frequency of infections or serious adverse events. Viral infections were numerically more frequent among elderly patients, whereas bacterial infections occurred slightly more often in younger patients. The overall incidence of adverse events remained low in both groups. This finding may reflect careful patient selection, regular monitoring, and cautious use of concomitant immunosuppressive therapies in routine clinical practice. Nevertheless, the relatively small number of elderly patients (n = 23) may have limited the statistical power to detect uncommon or delayed adverse events.

Clinical implications

With an aging RA population and growing hesitancy to prescribe biologics to elderly individuals, our findings provide reassuring short-term real-world evidence suggesting that RTX may be an appropriate therapeutic option for selected older adults with RA. Previous studies have suggested a corticosteroid-sparing effect of RTX and a potentially lower risk of tuberculosis reactivation or demyelinating events compared with TNF inhibitors; however, these outcomes were not specifically assessed in the present study. In clinical practice, RTX may nevertheless represent a useful therapeutic option for selected elderly patients with multiple comorbidities.

Strengths and limitations

The strengths of this study include its prospective design, the use of validated composite disease activity indices (DAS28-CRP, DAS28-ESR, SDAI, and HAQ), and the inclusion of a real-world cohort representative of routine clinical practice. In addition, efficacy and safety were evaluated using complementary clinical, biological, and patient-reported outcomes at predefined follow-up visits.

Several limitations should be considered when interpreting these findings. First, this was a single-center observational study including a relatively small number of elderly patients (n = 23). Because no formal sample size calculation was performed, the study was not statistically powered to detect small between-group differences or uncommon adverse events, nor was it designed to demonstrate equivalence between age groups. Consequently, the absence of statistically significant differences should not be interpreted as proof of equivalent efficacy or safety.

Second, patients who did not complete the six-month follow-up were excluded from the analysis, which may have introduced selection bias, particularly if treatment discontinuation was related to poor response or adverse events. Third, RTX treatment reflected routine clinical practice, resulting in some heterogeneity regarding the number of previous treatment courses and dosing regimens, which may have influenced treatment outcomes. Furthermore, serum Ig levels were not systematically monitored during follow-up, limiting a comprehensive assessment of hypogammaglobulinemia and its potential contribution to infectious complications.

Finally, the six-month follow-up provides information only on short-term efficacy and safety and does not allow assessment of long-term effectiveness, drug retention, delayed adverse events, or sustained remission. In addition, because of the observational design, residual confounding cannot be excluded, and causal inferences should be made with caution.

Despite these limitations, our findings are consistent with previously published registry and observational studies suggesting that RTX may be an effective treatment option in older patients with RA. Nevertheless, careful patient selection, appropriate vaccination according to current recommendations, and close monitoring for infectious complications remain essential in this population [24,25]. Larger multicenter studies with longer follow-up and adequate statistical power are warranted to confirm these findings and further characterize the long-term efficacy and safety of RTX in elderly patients with RA.

## Conclusions

The aging of the RA population requires an individualized approach to biologic therapy. In this prospective real-world study, RTX was associated with similar improvements in disease activity and functional outcomes over six months in elderly (≥65 years) and younger (<65 years) patients, with no statistically significant differences observed between the two groups. These findings provide reassuring short-term real-world evidence suggesting that RTX may be an appropriate therapeutic option for older adults with RA.

However, given the observational design, the limited number of elderly patients, and the absence of statistical power to demonstrate equivalence between age groups, these findings should be interpreted with caution. Treatment decisions should remain individualized, taking into account comorbidities, frailty, infection risk, and previous exposure to immunosuppressive therapies. Careful patient selection, appropriate vaccination, and close monitoring for infectious complications remain essential components of clinical management. Larger multicenter studies with longer follow-up are warranted to confirm these findings and further characterize the long-term efficacy and safety of RTX in elderly patients with RA.
